# Mining the multifunction of mucosal-associated invariant T cells in hematological malignancies and transplantation immunity: A promising hexagon soldier in immunomodulatory

**DOI:** 10.3389/fimmu.2022.931764

**Published:** 2022-08-16

**Authors:** Meng-Ge Gao, Xiao-Su Zhao

**Affiliations:** ^1^ Peking University People’s Hospital, Peking University Institute of Hematology, National Clinical Research Center for Hematologic Disease, Beijing Key Laboratory of Hematopoietic Stem Cell Transplantation, Beijing, China; ^2^ Research Unit of Key Technique for Diagnosis and Treatments of Hematologic Malignancies, Chinese Academy of Medical Sciences, Beijing, China; ^3^ Collaborative Innovation Center of Hematology, Peking University, Beijing, China

**Keywords:** mucosal-associated invariant T cells, transplantation immunity, hematological malignancies, immunotherapy, allogeneic hematopoietic stem cell transplantation (AlloHCT)

## Abstract

Mucosal-associated invariant T (MAIT) cells are evolutionarily conserved innate-like T cells capable of recognizing bacterial and fungal ligands derived from vitamin B biosynthesis. Under different stimulation conditions, MAIT cells can display different immune effector phenotypes, exerting immune regulation and anti-/protumor responses. Based on basic biological characteristics, including the enrichment of mucosal tissue, the secretion of mucosal repair protective factors (interleukin-17, *etc.*), and the activation of riboflavin metabolites by intestinal flora, MAIT cells may play an important role in the immune regulation effect of mucosal lesions or inflammation. At the same time, activated MAIT cells secrete granzyme B, perforin, interferon γ, and other toxic cytokines, which can mediate anti-tumor effects. In addition, since a variety of hematological malignancies express the targets of MAIT cell-specific effector molecules, MAIT cells are also a potentially attractive target for cell therapy or immunotherapy for hematological malignancies. In this review, we will provide an overview of MAIT research related to blood system diseases and discuss the possible immunomodulatory or anti-tumor roles that unique biological characteristics or effector phenotypes may play in hematological diseases.

## MAIT cell biological characteristics

Mucosal-associated invariant T (MAIT) cells are a group of unconventional T cells that are immunophenotyped as CD3+Vα7.2+CD161^hi^ cells, and CD8+CD161^hi^ cells account for more than 90% of MAIT cells (1–4). MAIT cells are tissue specific and enriched in mucosal tissues such as the lung and gut but are also present in other tissues, including the skin and liver (5–9). In humans, MAIT cells make up 1–10% of T lymphocytes in the blood, up to 10% of intestinal T cells, and up to 50% of all liver T cells (10). In human peripheral blood (PB), most MAIT cells express CD8 receptor (approximately 80% of the total MAIT), and a small portion express CD4 (less than 5% of the total MAIT) (10). Unlike traditional T cells, MAIT cells are not restricted by major histocompatibility complex (MHC) but recognize the MHC-related protein MR1. MAIT cells express a semi-invariant T cell receptor (TCR)-α chain (Vα7.2-Jα33/20/12 in humans) and a limited TCR-β chain reservoir, mainly from the TRBV20 and TRBV6 gene families (7, 11). They recognize riboflavin derivatives (5-OP-RU) synthesized by bacteria and yeast, and these metabolites bind to MR1 molecules (12, 13). MAIT cells can also be activated in the independent TCR pathway by proinflammatory cytokines such as interleukin (IL)-12/IL-18 (14–16). Upon activation, MAIT cells can respond rapidly to produce a series of cytokines, including interferon-γ (IFN-γ), tumor necrosis factor α (TNF-α), and IL-17 (17–19). The manner in which MAIT cells are activated results in distinct transcriptional programs. TCR-dependent activation results in an increased expression of retinoic acid-related orphan receptor γt (RORγt) (encoded by RORC) (19, 20), which is a unique property of the capacity to secrete molecules to mediate tissue repair (21). In TCR-independent activation, an elevated expression of T-bet (encoded by TBX21) has been shown, again consistent with mouse data showing a T-bet-associated IFN-γ bias (19, 20). In addition to cytokine production, the expression of granzyme B and perforin has been demonstrated to increase in activated MAIT cells, thereby enhancing their cytotoxic capacity  (22). These characteristics make MAIT cells have only anti-bacterial and immunomodulatory activities but may also affect the occurrence and development of tumors.

## The immune regulation of MAIT cells in hematopoietic stem cell transplantation

Currently, MAIT cells are not well studied in the hematological system. Due to the unique biological characteristics and multiple immune roles, blood-related studies of MAIT cells have focused mainly on immune regulation in the allogeneic hematopoietic stem cell transplantation (allo-HSCT) setting and anti-/protumors in hematological malignancies. Allo-HSCT is an effective, and even the only, treatment for hematological malignancies such as leukemia. Severe graft-*versus*-host disease (GVHD) and relapse after transplantation are the main reasons leading to transplantation failure and affecting the survival of patients (23–27). One of the main factors in the induction of GVHD is the overactivation of certain T cell subsets from donors, but excessive immunosuppression leads to an increased risk of infection and relapse (23–25). At present, the prevention and the treatment of acute GVHD involve mainly inhibiting the activation of T cells or upregulating the proportion of certain cell subsets with immunomodulatory effects, such as regulatory T (Treg) cells (28, 29) and myeloid-derived suppressor cells (MDSCs) (30). Since anti-GVHD and anti-tumor treatments are often contradictory in clinical practice, around allo-HSCT, the main hotspots of current basic and clinical translational medicine have focused on how to induce appropriate immune tolerance after HSCT to reduce the incidence of GVHD while preserving or even enhancing graft-*versus*-leukemia (GVL) effects. Most of the previous related studies had not been able to well separate the similar alloimmune reactivity behind the anti-GVHD and GVL effects, while MAIT cells produce a variety of cytokines after being activated by different stimulatory pathways of cytokines or TCR signals and participate in immune regulation and anti-tumor immunity at the same time, which may provide a new intervention strategy for the clinical prevention and treatment of GVHD and leukemia relapse.

### Reconstitution of MAIT cells after transplantation

MAIT cells reconstituted slowly after HSCT. MAIT absolute cell counts in blood dropped to a nadir on the day of https://www.sciencedirect.com/topics/medicine-and-dentistry/peripheral-blood-stem-celll transplantation (PBSCT), followed by early and rapid recovery to a plateau from day 30 to day 100 after https://www.sciencedirect.com/topics/medicine-and-dentistry/hematopoietic-cell (31), and remained lower than the MAIT cells of healthy donors for at least 1 year after HSCT (14, 31). MAIT cell reconstitution correlated significantly with age (32, 33) and cell source (32, 34–37). Since the umbilical cord blood (UCB) contains much lower frequencies of MAIT cells compared with adult graft sources (35), compared with PBSCT recipients, the recovery of MAIT cells in recipients infused with UCB grafts was highly impaired within 1 year after HSCT (31, 32, 34), and normal values after UCB transplant were reached at approximately 5 years in children (34) and approximately 10 years in adults (32). Regarding the effect of conditioning regimens on reconstitution, Bhattacharyya A et al. found no differences in early or late post-transplant MAIT cell reconstitution in patients receiving myeloablative (MA) or nonmyeloablative conditioning (31). In contrast, Solders M et al. showed that patients without anti-thymocyte globulin as well as patients conditioned with MA conditioning rather than reduced intensity conditioning had significantly higher MAIT cell frequencies (38). Notably, the type of transplantation under different transplantation settings affected the reconstitution of MAIT cells after transplantation. Under the “Beijing Protocol” transplantation settings in our institute (14), the number of MAIT cells in haploidentical HSCT patients was significantly lower than the number of MAIT cells in sibling-identical HSCT patients within 180 days after transplantation, and the difference in reconstitution between the two groups gradually decreased at 180 days post-transplant (14). In the transplantation setting of Bhattacharyya A *et al.* (31), patients who received PBSC transplant with post-HSCT cyclophosphamide (Cy) had poor MAIT cell recovery compared with the recipients of PBSC grafts without post-HSCT Cy. Other factors in previous studies, such as total body irradiation (31), glucocorticoids and calcineurin inhibitors (32), HLA match/mismatch, and indication for transplantation (acute leukemia compared to other diagnoses), were not found to affect the reconstitution of MAIT cells after HSCT. Additionally, the rapid reconstitution of MAIT cells after transplantation was related to the increase in the abundance of intestinal flora (such as *Blautia* and *Bifidobacterium*) (14, 31, 32, 39–41), which may be due to the destruction of the intestinal mucosal barrier by pretransplant pretreatment with cytotoxic drugs, resulting in the increased permeability of the intestinal epithelium that allows intestinal bacterial antigens to contact and activate (by the MR1/TCR-dependent pathway) MAIT cells from grafts, which may also be the reason for the rapid proliferation of MAIT cells within 30 days post-transplant (14).

Taken together, MAIT cell reconstitution depends on factors such as age, cell source, conditioning regimens, transplant types, gut microbiota, and immunosuppression. It should be noted that, in the transplantation settings of the above-mentioned studies, the transplant grafts included bone marrow stem cells, PBSCs, or UCB, and the conditioning regimens were not uniform, which may lead to differences in the study results. Currently, no studies have focused on the effect of post-transplant infection (bacteria and viruses, such as cytomegalovirus and Epstein–Barr virus) on MAIT cell reconstitution. Furthermore, proinflammatory signals induced by immunosuppressive therapy (4, 11, 36), along with an altered gut microbiota composition caused by conditioning therapy, as well as altered dietary intake and antibiotic use (36, 42) may further influence MAIT cell reconstitution and function after allo-HSCT.

### Effect of MAIT cells on graft-*versus*-host disease

The anti-GVHD effect of MAIT cells has been well established in several studies (**Table 1**). Kawaguchi K et al. demonstrated that MAIT cell count on day 60 after allo-HSCT was the only independent risk factor for grades I–IV and II–IV acute GVHD (33). Other studies have shown that the decreased proportion of peripheral CD161^hi^CD8+ T and MAIT cells may be associated with acute and chronic GVHD (8, 43). A possible explanation could be that, under the action of chemokines, CD161^hi^CD8+ T and MAIT cells were recruited to inflammatory cells or lesions. Among CD8+ T cells, the tissue homing properties of subsets expressing CD161 have been well defined (44, 45). CD161^hi^CD8+ MAIT cells are highly enriched in mucosal tissues and significantly upregulate chemokine receptors such as CXCR6 and CCR6 (17), and CD161 and CCR6 alone favor T cell migration and tissue homing (44, 46). In the intestinal mucosa of acute GVHD patients, the absolute number of T helper (Th) 17 cells of CD161, RORγt, and CCR6 was significantly higher (43). Furthermore, CD8+ T cells expressing intermediate and high levels of CD161 secreted high levels of IL-22, a cytokine involved in tissue repair and epithelial defense (44). Another recent study showed that high MAIT cell counts in infused grafts were associated with a lower incidence of gut acute GVHD after allo-HSCT and that MAIT cell counts in infused grafts could affect the abundance and composition of gut microbiota early after transplantation (14). *In vitro* studies have shown that MAIT cells can transform into MAIT17 subsets or secrete increased IL-17 upon stimulation with TCR-specific (riboflavin metabolite 5-OP-RU or *E. coli*) or nonspecific signals (CD3/CD28) (14, 20, 47, 48). IL-17 has been shown to play an important role in maintaining the integrity of the intestinal mucosa (14, 26, 49–51). RNA-seq technology analysis also confirmed that, under TCR stimulation, upregulated IL-17F expression and a large number of genes associated with tissue repair characteristics were observed, including *Furin*, *TNF*, *CSF1*, and *CCL3* and other genes as well as various growth factors (21, 52). In an *in vitro* wound-healing assay, MR1 blockade abrogated the effect, confirming the TCR-dependent tissue repair potential of MAIT cells (21, 44–48) and demonstrating that TCR-dependent activation was essential for the expression of tissue repair-associated molecules by MAIT cells.

**Table 1:** Summary of research in graft-*versus*-host disease.

GVHD types	Definition of MAIT cells	MAIT or MAIT subsets Frequencies	MAIT cell cytokines or effector phenotypes	Flora composition	References
Acute GVHD Chronic GVHD		↓CD161^hi^CD8+ and CD161+CD4+ in periphery	↓IFN-γ, ↓IL-17		(8, 43)
Acute GVHD		↓CD8+CD161^hi^ in graft PB			(8)
Acute GVHD	CD45+CD3+CD161^++hi^Vα7.2+ CD3+CD8+CD161^++++hi^Vα7.2+	↓MAIT cells in the early post-HCT period		Higher abundance of *Blautia spp.* and *Bifidobacterium longum* associated with higher MAIT cell counts in blood	(31)
Acute GVHD	CD161^hi^TCRVα7.2+CD3+	↓MAIT cells (0.48/μl) in periphery on day 60 post-HSCT			(33)
Gut acute GVHD	CD3+CD161^hi^ Vα7.2+	↓MAIT cells in grafts and in the periphery of early post-HCT period ↑CD161^hi^CD8+ cells in the lesion sites of gut aGVHD ↑Rorγt+MAIT (MAIT17) cells, ↑CD4-CD8-MAIT cells	↑CD69, ↑CXCR3, ↑CXCR4, ↑Rorγt, ↑T-bet in periphery	Gut aGVHD-associated flora: *Enterococcus*, *Streptococcus*, *Flavobacteriales*, *Lactobacillus*, *Firmicutes*	(14)
Chronic GVHD	CD3+CD161^hi^ Vα7.2+	↓MAIT cells in periphery	No change: granzyme B, IFN-γ, and IL-17 ↑Rorγt in CD8+CD161+ T cells	The riboflavin pathway of microbiomes correlated with MAIT cell reconstitution	(32)
Acute GVHD	CD3+CD161^hi^ Vα7.2+	MAIT cell frequency did not correlate with GVHD status following HSCT ↑CD8+MAIT cells frequency			(38)

+, Positive; +++, Strong positive or high expression; ↑, Upregulated expression or secretion; ↓, Downregulated expression or secretion.

Studies have shown that the flora associated with the occurrence of gut acute GVHD includes *Enterococcus*, *Streptococcus*, *Flavobacteriales*, *Lactobacillus*, and *Firmicutes* (14). Notably, *Enterococcus*, *Streptococcus*, and *Lactobacillus* were impaired in riboflavin biosynthesis. *Bacteroidetes*, *Proteobacteria*, *Actinobacteria*, and *Firmicutes* have recently been shown to activate MAIT cells in decreasing order (53–55). A recent study by Andrlová H *et al.* also showed that a higher abundance of *Bacteroidetes* in the early post-transplant period was associated with a higher proportion of MAIT cells and favorable transplantation outcomes, and specific bacterial taxa and their riboflavin synthesis pathway genes or key enzymes supported MAIT cell reconstitution (56). The reason for this result might be that, on the one hand, these gut microbiota with nonriboflavin metabolic pathways cannot effectively activate intestinal MAIT cells, especially the MAIT17 subsets, so that the intestinal protective cytokines or barriers against inflammation are reduced, leading to the occurrence of gut acute GVHD (14). On the other hand, a non-efficient or low-efficiency riboflavin biosynthetic pathway allows these bacteria to escape MAIT cell-mediated host detection and enhance their pathogenicity (14, 55).

Of note is that most studies have focused on donor-derived MAIT cells affecting GVHD development and progression by affecting immune reconstitution after transplantation or by interacting with gut microbiota. However, it was shown for the first time that, in MR1-/- and IL-17A-/- mouse transplant models, MAIT cells from the recipient but not the donor after bone marrow (BM) transplantation produced a large amount of IL-17A to promote gastrointestinal integrity, modulate microbial communities, and inhibit alloantigen presentation and effector T cell expansion, inhibiting the occurrence of GVHD (51). However, for human allo-HSCT, whether donor-derived MAIT cells or recipient-derived MAIT cells affect the occurrence of GVHD or whether both donor-derived and recipient-derived MAIT cells play different anti-GVHD leading roles at different reconstruction stages after HSCT remains to be further elucidated.

Furthermore, activated MAIT cells can inhibit the proliferation of CD4+ T cells (14, 31). The possible reason for this result is that, under specific activation conditions, MAIT cells can express higher levels of inhibitory molecules such as *PD-1*, *CTLA-4*, and *TIM-3* (50, 57, 58). The engagement of these molecules with their respective ligands results in the inhibition of T cell responses (59). Another possible explanation is that MAIT cells express immune regulation/suppression-related genes, such as *DUSP2*, *SOCS3*, and *ZFP36*, and express *RUNX3*. *PRDM1* defines the ontogeny and activation of conventional T cell lineages (47, 50), which determines the secretion of inhibitory cytokines, such as IL-10 and IL-4, by regulating cytokine signaling and ultimately inhibiting the proliferation of effector T cells. Of course, more research data are needed to support these findings. In the MR1-/- mouse model, recipient MAIT cells were found to have the ability to inhibit alloantigen presentation by donor dendritic cells (DCs) and the subsequent expansion of effector T cells following transplantation, culminating in the attenuation of GVHD (51). These data suggest that MAIT cells suppress effector T cells by downregulating the function of antigen-presenting cells.

In short, MAIT cells are rapidly activated and proliferate under stimulation of the intestinal flora (MR1/TCR-dependent pathway) and cytokines (non-TCR-dependent pathway). Activated MAIT cells, in turn, can inhibit the occurrence of gut acute GVHD by exerting immunosuppressive effects, expressing intestinal mucosal protective cytokines, and regulating the intestinal flora (14). Nevertheless, there is still a lack of research on whether MAIT cells can have a stable GVL effect in the transplantation setting. In different clinical backgrounds, the distribution of MAIT cell subsets with different phenotypes or expressing different transcription factors in homeostatic or pathological states, their activation states to different antigenic stimuli, and their immune functions may be different. In the process of allo-HSCT, how different MAIT cell subsets play a role in immune regulation or anti-leukemia remains to be further clarified.

### Effects of granulocyte colony-stimulating factor mobilization on MAIT cells before transplantation

Granulocyte-colony stimulating factor (G-CSF) has been widely used to mobilize bone marrow hematopoietic stem/progenitor cells for transplantation in the treatment of hematological malignancies. *In vivo*, G-CSF can affect the differentiation and activation of specific lymphocyte subsets and induce the preferential mobilization of naive T cells and immune tolerance (25, 30, 60–63). G-CSF mobilization has been demonstrated to reduce GVHD with preservation of the GVL effect (25, 64–66). A growing body of studies confirmed that G-CSF could attenuate the reactivity of T and natural killer (NK) cells by inducing Th2 cell polarization (62, 67) and promoting the generation of Treg cells (64, 68), tolerogenic DCs (25, 64), and possibly MDSCs (30). There are limited reports on the effect of G-CSF on MAIT cells. The proportion and number of MAIT cells in donor grafts did not appear to be affected by G-CSF mobilization (69). Moreover, a greater fraction of IL-17-secreted CD8+CD161^hi^ was found in adult blood following G-CSF mobilization (35). MAIT cells (CD3+CD161+Vα7.2TCR+) were the only CD8+ IL-17A-secreting T cell subset following G-CSF mobilization, and the proportions of RORγt-expressing or coexpressing IFN-γ/IL-17A associated with chronic GVHD in MAIT cells were further enhanced with G-CSF mobilization (69). These results also suggested that G-CSF mobilization did not affect or even strengthen the regulation of TCR signaling in MAIT cells.

Overall, MAIT cells are a class of cells with proinflammatory, anti-tumor and immunomodulatory effects, and the number and function of each subset after G-CSF mobilization have important implications for the prognosis of transplantation. However, the effects of G-CSF mobilization on the differentiation of MAIT cells and their subsets, the distribution of surface receptors or effector phenotypes, and the exertion of different effector functions (anti-GVHD and GVL effects) remain to be further elucidated.

## MAIT cells and hematological malignancies

MAIT cells have been detected in a variety of human tumor types, such as colorectal (70, 71), cervical (72), lung (73, 74), liver (75), and kidney cancers (76). Cancer has an effect on MAIT cell frequency, phenotype, and function, whereas the effect of MAIT cells on cancer may vary greatly from cancer to cancer (77). Collectively, no conclusion has been drawn on whether MAIT cells play an anti-tumor or a tumor-promoting role in different tumors or microenvironments. The potential role of MAIT cells in hematological malignancies has also not been well described. MAIT cells have unique effector phenotypes (78), which not only facilitates the best identification of MAIT cells and all conventional nonMAIT cells (such as conventional CD8+ T cells) but also confers unique multiple immune roles to MAIT cells in various pathological conditions.

### Potential role of MR1 molecular mediation in hematological malignancies

Major histocompatibility complex class 1-related gene protein (MR1) is a monomorphic antigen-presenting molecule (**Table 2**). The default extracellular expression of MR1 is very low in the absence of its ligands and is upregulated under inflammatory condition activation (4, 79, 80). The primary role of MR1 is to present the conserved ligands of microbial metabolites to MAIT cells (most effective ligand 5-OP-RU and 5-OE-RU) (69). MR1 molecules were found on several immune cell types, such as monocytes and B cells (17, 21, 81). Furthermore, MAIT cells were activated by B cells infected with various bacterial strains but not by uninfected cells (81). Accordingly, MAIT cells may be involved in the occurrence and development of malignant hematological tumors through MR1 molecules on monocytes or B cell-related tumor cells. Interestingly, the expression of MR1 molecules was found in multiple myeloma (MM) cells and leukemia cell lines such as THP-1 and K562. MAIT cells have a certain killing function on these tumor cells *in vitro* (79, 86). Crowther *et al.* demonstrated that a human T cell clone potentially recognizes a specific cancer or associated metabolite, restricted to MR1, and mediates the lysis of different types of cancer cells, including leukemic cell lineages; as such, the human T cell clone mediated *in vivo* leukemia regression and conferred longer survival in mice (99, 100). These results suggested that MAIT cells may identify and kill MR1-expressing malignant tumor cells in MR1-dependent methods and that MR1 may become an attractive target for future treatment. In contrast, a recent study in MR1^-/-^ mice found that MAIT cells promoted tumorigenesis, growth, and metastasis through melanoma tumor MR1 (13). Thus, whether the MR1 pathway of MAIT cells mediates protumor or anti-tumor effects is still inconclusive, and the role of MR1 in malignant hematological tumors still needs more research to be confirmed.

**Table 2:** Major effector phenotypes of MAIT cells.

Phenotypes	Distribution in MAIT cells	Ligands/substrates	Ligand distribution	Functions	References
MR1	MAIT cells	5-OP-RU/5-OE-RU	Vitamin B metabolites synthesized by bacteria and yeast	Presents microbial metabolites to MAIT cells that can be used to recognize and activate MAIT	(4, 79–81)
CD161 (encoded by KLRB1)	Mature MAIT cells, downregulated expression of activated MAIT cells	LLT1 (encoded by CLEC2D)	Mature dendritic cell, plasmacytoid dendritic cell, macrophages, B cells, NK cells, T cells Tumor cell included NHL	a) Expression of CD161 is associated with good prognosis in most cancers b) Engagement on NK cells trigger inhibition c) LLT1/CD161 interaction modulates immune responses and other unspecified effects	(44, 82–85)
MDR-1 (encoded by ABCB1)	CD8+CD161^hi^ or IL-18Rαhi CD161^hi^ CD4-CD161 ++vα7.2+	A variety of different substrates, including cyclosporin A and verapamil		a) Resistance to certain chemotherapy/cytotoxic drugs b) Protection from endogenous metabolites or xenobiotics that may be secreted by gut bacteria	(17, 43, 58, 86–89)
FasL	MAIT cells	Fas	Various tumor cells	Initiate tumor cell apoptosis	(20, 90)
CCR6	MAIT17 cells	CCL20	The liver, colon, small intestine, lung, and skin	a) Recruit blood MAIT cells to sites of inflammation b) Associated with chronic GVHD	(43, 91)
CCL3, CCL4, CCL5	RORγt+MAIT (MAIT17) cells			a) Tissue repair b) Recruit NK cells, macrophages, neutrophils, eosinophils, DCs, and conventional T cells	(43, 58, 88)
PD-1	Activated MAIT cells	PD-1L	Tumor cells	a) An immune-inhibitory receptor expressed in activated T cells b) Involved in the regulation of T cell functions	(92–96)
ICOS	RORγt+MAIT (MAIT17) cells			Optimal activation and maintenance of RORγt expression	(97)
CCR7, CD62L	CD4+MAIT cells Immature MAIT cells			a) Regulating MAIT cell development b) CCR7 is selectively required for the differentiation of Rort+MAIT17 subset c) Effector memory-like CCR7- CD62Llow	(57, 78, 91)
CD27, CD45RO, CD44	Effector memory MAIT cells			Associated with MAIT cell development and effector memory phenotype	(57, 78, 91)
IL-7R (CD127)	RORγt+MAIT cells			a) IL-7 enhances MAIT cell responses to bacteria and promotes cytotoxicity b) Enhanced production of IL-17A by MAIT cells	(57, 91, 98)
IL-12R, IL-18R	CD8+MAIT cells			a) IL-12 and IL-18 potentiate MR1-dependent bacterial MAIT cell activation b) IL-12 is particularly important for IFN-γ production	(57, 91, 98)

### Potential role of CD161-LLT1 mediation in hematological malignancies

The natural cytotoxicity receptor CD161 (NK1.1 in mice) is generally expressed in NK cells and 24% of T cells including both γδ and αβ TCR-expressing subsets, natural killer T cells (NKT), MAIT cells, monocytes, and DCs (44, 82, 101). The single CLEC2D gene encoding LLT1 is identified as a ligand of CD161 (82, 101). Restricted to hematopoietic cells, LLT1 is not expressed on the surface of resting PBMCs but can be transiently expressed on activated B cells, dendritic cells, T cells, and NK cells (44, 82, 83). Some tumors of hematopoietic origin are also detected by LLT1 expression (75), which is highly expressed by germinal center (GC) B cells and is maintained in the group of non-Hodgkin’s lymphomas that derive from GC B cells (82, 83). These include Burkitt lymphomas, follicular lymphomas, and GC-derived diffuse large B cell lymphomas (82, 83). In addition, Freeman G *et al.* reported LLT1 on nodular lymphocyte-predominant Hodgkin lymphomas, and LLT1 triggering may play a key role in GC reactions, promoting B cell activation and the downregulation of CXCR4 (84). Significantly, the interaction of LLT1 with the CD161 receptor is described as inhibitory in NK cells that inhibits their cytotoxicity and cytokine secretion (44, 83). The blocking of LLT1-CD161 restored the function of NK cells (83). In addition, CD161 receptor engagement with the ligand LLT1 was not sufficient to trigger IFN-γ production among T cells unless simultaneously engaged with CD3 (101). LLT1 interaction with CD161 did not modulate degranulation in CD8 T cells but partially inhibited TNF-α production (85). In short, the current findings strongly suggest that LLT1-CD161 can modulate NK and T cell responses. Nevertheless, no studies have explored the functional roles triggered by CD161-LLT1 between MAIT cells and hematological tumors. In the future, additional studies will be required to better understand the true consequences of this ligand/receptor interaction (44, 101). The blocking or enhancing of the interaction of LLT1/CD161 with anti-LLT1 monoclonal antibodies to enhance antitumor NK and T cell (including MAIT cells) activity may become a potential therapeutic approach.

### Potential role of other MAIT unique effect phenotypes in hematological malignancies

The cassette-multi-drug efflux protein 1 (MDR1), another high-expression molecule of MAIT cells encoded by *ABCB1*, is the prototypical drug efflux pump that has been described to mediate multi-drug resistance in various malignant cells (87, 102). MDR-1 has extensive specificity for various substrates, including those that also inhibit transport, such as cyclosporin A and verapamil (87). This is beneficial for MAIT cells to become the optimal survival cell subpopulation in chemotherapy. Interestingly, MAIT cells can also express the FasL/sFasL death ligands. Both TCR and cytokine-activated MAIT cells can rapidly upregulate the FASLG (FasL) gene expression (20). It has been reported that the antigen-specific cytotoxicity of iNKT cells *in vivo* almost entirely depends on the interaction between CD95 (Fas) and CD178 (FasL), and this mechanism can be effectively used for anti-tumor reaction (90). Therefore, in addition to the cytotoxic effects such as perforin/granzyme and IFN-γ, whether MAIT cells can also exert anti-tumor effects through the Fas/FasL pathway remains to be further elucidated. With the high expression of IL-7R, IL-12R, IL-18R, and other receptors (**Table 2**), cytokines can directly stimulate MAIT cells to produce IFN-γ and release granzyme B and perforin (57, 91, 97, 98). In principle, these specific phenotypes or targets are beneficial for MAIT cells to play a potential anti-tumor effect in hematologic malignancies, which may facilitate the use of MAIT as a candidate subset for immunotherapy in hematological malignancies.

### Related research on MAIT cells in clinical hematological malignancies

To date, the involvement of MAIT cells in hematological malignancies, especially leukemia, remains largely unexplored. MM is a hematological malignancy characterized by the uncontrolled growth of plasma cells from the BM (99). In patients with newly diagnosed or untreated MM, the frequency of MAIT cells was significantly reduced, especially the CD8+ and CD8-CD4- subsets (86, 92). The MAIT1 subset in newly diagnosed or untreated MM patients was dysfunctional, with reduced IFN-γ (and TNF-α) production, but the ability to produce IFN-γ appeared to be restored in samples from relapsed/refractory MM patients (86, 92, 99). The exact role MAIT cells play in MM remains unclear, but the authors did demonstrate that MAIT cells were capable of killing myeloma cell lines, suggesting the potential for harnessing MAIT cells as an immunotherapy (77, 86). A recent prospective study including 216 cases of acute myeloid leukemia (AML) showed that the number of MAIT cells in PB from newly diagnosed AML was significantly reduced, and the degree of reduction was associated with a high-risk cytogenetic karyotype and IDH1/2 mutation, suggesting that the loss of MAIT cell number or function may be associated with AML disease progression (103). Another study by Wallace ME *et al.* observed deficiencies in MAIT cells in patients with chronic lymphocytic leukemia (CLL) (104), but the authors did not indicate a causal relationship between MAIT cell deficiency and the pathogenesis of CLL or the possible mechanisms involved. CLL is the malignancy of mature B cells; in the context of CLL, B cells can act as antigen-presenting cells in MAIT responses to intestinal microbes, and bacterial infection is associated with increased MR1 expression on B cells (81, 104). While it is far from certain whether MAIT cells prove to be important in CLL, the relationship between MAIT cells and CLL and the mechanism of action deserve further investigation. Moreover, a rare case of peripheral T cell lymphoma caused by MAIT cells has been described, but the effector function or mechanism of MAIT cells has not been further explored (105). *In vitro* assays confirmed that MAIT cells isolated from PB from healthy individuals not only had lymphokine-activated killing activity but also exhibited direct cytotoxicity in the K562 cell line *via* the degranulation of granzyme B and perforin (106).

**Figure 1:**
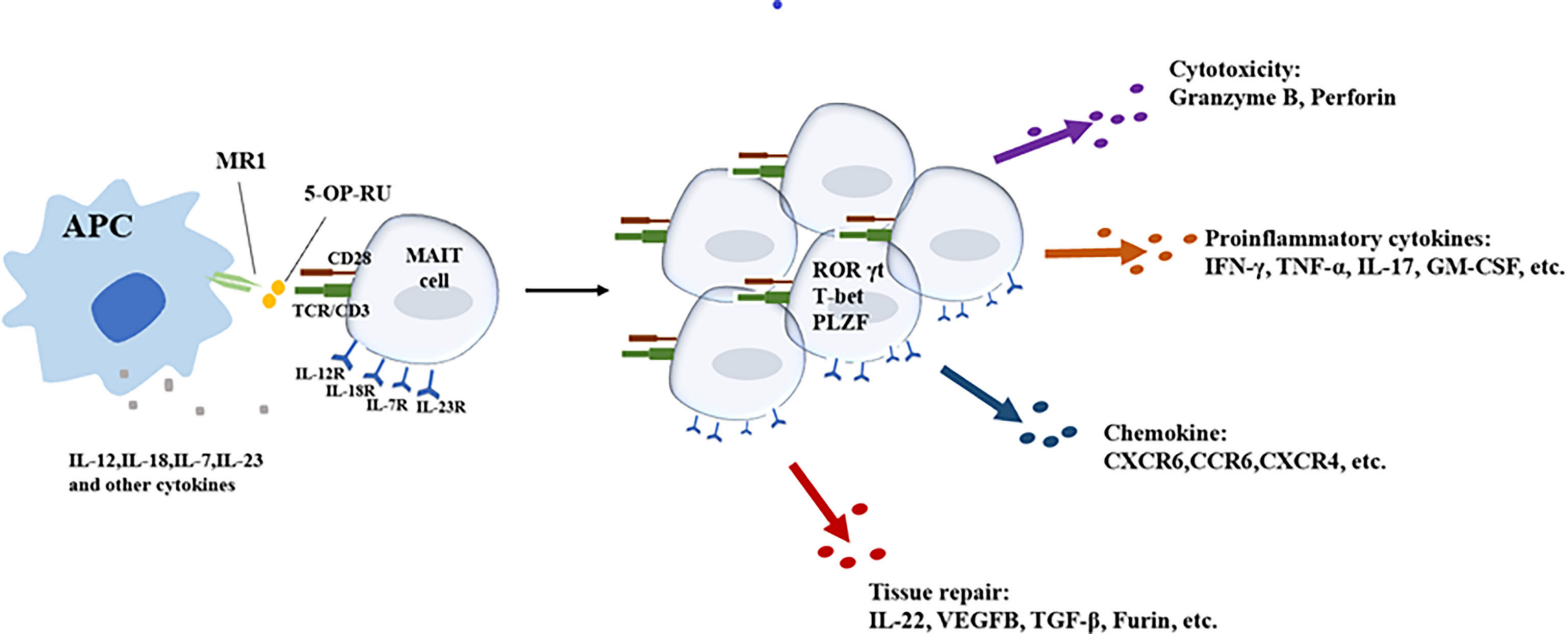
Different activation pathways of MAIT cells and main effector functions of activated MAIT cells.

In short, most of the above-mentioned studies (**Table 3**) have observed the effects of blood diseases on the frequency and function of MAIT cells, but whether MAIT cells affect the occurrence and development of blood diseases, especially malignant tumors, and the mechanism of the effects have not been well described. The role of specific targets or effector phenotypes of MAIT cells in hematological malignancies deserves further investigation (**Figure 1**).

**Table 3:** Summary of research in hematological malignancies.

Disease types	Method of MAIT detection	MAIT cell frequencies	MAIT cell cytokines or effector phenotypes	MAIT subset frequencies	References
MM	MR1–5-OP-RU tetramers+ TRAV1-2+	↓ in periphery	↓IFN-γ, ↓CD27	↓CD8+, ↑CD4, ↑DN	(86)
MM	CD3+ CD161+Vα7.2	↓in periphery and bone marrow	↓IFN-γ, ↓TNFα	↓CD8+, ↓DN, unchanged CD4+	(92)
AML	CD3+CD8+CD161^hi^Vα7.2	↓ in periphery			(103)
CLL		↓			(104)

↑, Upregulated expression or secretion; ↓, Downregulated expression or secretion.

## MAIT cells in chemotherapy and immunotherapy

### Effect of MDR-1 expression on chemotherapy in MAIT cells

The high expression of ATP-binding MDR1 is a striking feature of MAIT cells (17, 58). Anthracyclines act as one of the major substrates of the resistance transporter ABCB1 (also known as MDR1), which also explains the significantly higher resistance of MAIT cells to anthracycline (daunorubicin) cytotoxic drugs compared to other CD8+ T cell subsets (17, 87). In AML patients, the high ABCB1-mediated drug efflux capacity of the IL-18Rα^hi^ CD161^hi^ T cell subset conferred resistance to anthracycline chemotherapy (88). Paradoxically, Comont T *et al.* observed that MAIT cells were highly sensitive to AML chemotherapy (including azacytidine, idarubicin, *etc.*) and were depleted from the circulation during the induction treatment (103). Novak et al. explored MAIT cells in PB from patients with hematological malignancies who received a course of MA conditioning (*e*.*g*., with a combination of carmustin, etoposide, cytarabine, and melphalan) before autologous CD34 stem cell transplantation (89) and found that a high proportion of MAIT cells survive myeloablative chemotherapy and maintain their capacity to fight against infections, probably on mucosal surfaces (89). Theoretically, the high expression of MDR1 in MAIT cells can indeed confer certain resistance to certain chemotherapeutic drugs in MAIT cells, but the current studies are limited, and the conflicting conclusions prompt the need for stronger data to further support this hypothesis. Furthermore, in the chemotherapy of hematological malignancies, it is also worthwhile to continue to explore the specific environment or stimulation conditions to increase the expression of MDR1 in MAIT cells to enhance the drug resistance of MAIT cells while maintaining their anti-tumor effector functions.

### MAIT cells and immunotherapy

MAIT cells express many targets of immune checkpoint inhibitors, highlighting the potential importance of these cells in immune checkpoint therapy. PD-1 is a well-known target of immune checkpoint inhibition in cancer, as tumor cells are able to evade the immune system through PD-1-PDL1/2 signaling (92). MAIT cells express PD-1 in both blood and peripheral sites (93, 94), and enhanced PD-1 expression has been shown on CD4+ and CD8+ T cells in some cancer patients and other disease settings (58, 95). PD-1 levels were increased in MAIT cells in the BM and PB of patients compared with healthy controls, and *in vitro*/*in vivo* PD-1 blockade experiments demonstrated the successful reactivation of MAIT cells and a significant reduction in mouse tumor burden (92). The impact of MAIT cells in the MM microenvironment as well as the improvement of their effector functions through immune checkpoint blockade represents a relevant and attractive field for immune monitoring and immunotherapy in MM. A recent study showed that, in melanoma patients treated with anti-PD-1 therapy, patients with higher MAIT cell counts had a higher response rate to treatment, and anti-PD-1 therapy increased the expression of cytotoxic effect-related genes in tumor-infiltrating MAIT cells (96), suggesting that some treatments may have an anti-tumor effect by promoting the immune activation and killing function of MAIT cells. In addition, *Bifidobacterium longum* was particularly related to higher MAIT cell counts in the blood and recovery of MAIT cells after transplantation (31). Interestingly, in studies investigating the efficacy of anti-PD-L1 therapy, *Bifidobacterium* was significantly associated with anti-tumor effects and was most abundant in patients who responded to anti-PD-1 therapy (37), also providing the possibility for future microbiota transplantation by increasing the frequency and activation of MAIT cells or enhancing the efficacy of immune checkpoint inhibitors.

MR1 is also an attractive target for future therapy. The singlet receptor MR1 is highly conserved among individuals and binds predominantly to MAIT cells, eliminating the need to design new TCRs for each patient with different cancer types or HLA alleles and natural tropism for specific tissues, which can easily target mucosal tissues such as the liver and gut. Furthermore, nonMAIT, MR1-restricted T cells have recently been shown to recognize and kill several tumor cells in an MR1-dependent manner (58, 100). However, it is currently unclear whether this tumor-derived compound also contains MAIT cell antigens (58, 100). Regulation of MR1 expression is currently not well described in healthy tissues or tumor cells. Since MAIT cells are highly competent cytotoxic cells with distinct tissue propensities, redirecting these functions to other antigens may provide new therapeutic approaches for difficult-to-treat hematological malignancies.

In addition to therapies using MAIT cells to directly target tumors through their endogenous TCRs, MAIT cells may be an ideal host for chimeric antigen receptor (CAR)-T cell therapies (58, 107). The treatment of hematological malignancies by autologous T cells expressing CAR is a breakthrough in the field of cancer immunotherapy. Since they are not selected by classical MHC/peptide complexes and express semi-invariant T cell receptors, MAIT cells do not mediate allogeneic activity, prompting their use as a new source of universal effector cells for allogeneic CAR-T cell therapy without inactivating its endogenous TCR (108). In the latest study, researchers targeted tumor-associated macrophages by mesothelin-targeting CAR (MCAR)-engineered MAIT (MCAR-MAIT) cells and found that the targeting and killing of tumor-associated macrophages by MCAR-MAIT cells may be the reason for their persistent tumor-killing ability and activation (109), supporting the human cancer therapeutic potential of CAR-MAIT cells. In addition, another study reported the viability of CD19-CAR MAIT cells (108), demonstrating their anti-tumor efficacy *in vitro* and their ability to engraft without mediating https://www.sciencedirect.com/topics/medicine-and-dentistry/graft-versus-host-disease in preclinical immunodeficient mouse models, thus having the potential to provide a suitable alternative to current autologous CAR-T cells to treat patients regardless of HLA disparity. Collectively, the immunotherapy potential of MAIT cells is still theoretically feasible, and more robust data or studies are needed to establish the feasibility and reliability of MAIT cells for clinical cell therapy or immunotherapy methods.

## Conclusions and prospects

Currently, the limited research on MAIT cells in hematological diseases has focused on two areas: malignant tumors and transplantation immunity. First, in terms of hematological malignancies, the current studies are inconclusive about what role MAIT cells play in different tumors. In this regard, future studies need to confirm, on the one hand, the functional effects of MAIT cells or subsets on different hematological tumors and the potential specific mechanisms of these effects. On the other hand, as an attractive target of MAIT cells, MR1 needs to be better explored for the mechanisms of hematological disease-associated antigens and antigen/MR1 complexes presented in MR1 to successfully target MAIT cells to tumors. Additionally, noteworthy is the fact that MAIT cells may be ideal hosts for CAR-T cell therapy and may provide new and effective treatments for hematological malignancies. Second, for the field of transplantation immunity, the anti-GVHD effect of MAIT cells has been well described. However, MAIT cells are also heterogeneous, with different cell subsets possibly having different functions, and there may be many MAIT cell subsets that have not yet been discovered or fully elucidated. Therefore, future studies will provide stronger evidence for the regulatory effect of MAIT cells on GVHD. Moreover, it is necessary to clarify the heterogeneity of MAIT cells and the distribution and function of each subset *in vivo* under different activation conditions during transplantation and further confirm the role of specific subsets of MAIT cells in anti-GVHD immune regulation and anti-leukemia to ensure that only the optimal cells are transferred. These findings will provide a new intervention strategy for the clinical prevention and treatment of GVHD and leukemia relapse and further improve the efficacy of transplantation.

## Data availability statement

The original contributions presented in the study are included in the article/supplementary material. Further inquiries can be directed to the corresponding author.

## Author contributions

M-GG and X-SZ jointly conceived the article. M-GG conducted the literature review and drafted the manuscript. X-SZ modified the review. All authors contributed to the article and approved the submitted version.

## Funding

The work was supported by the National Key Research and Development Program of China (2021YFC2500300), the National Natural Science Foundation of China (grant no. 81870137), and Innovative Research Groups of the National Natural Science Foundation of China (grant no. 81621001) and CAMS Innovation Fund for Medical Science (grant number: 2019-12M-5-034).

## Conflict of interest

The authors declare that the research was conducted in the absence of any commercial or financial relationships that could be construed as a potential conflict of interest.

## Publisher’s note

All claims expressed in this article are solely those of the authors and do not necessarily represent those of their affiliated organizations, or those of the publisher, the editors and the reviewers. Any product that may be evaluated in this article, or claim that may be made by its manufacturer, is not guaranteed or endorsed by the publisher.
